# Tribological Failure in Modern Total Hip Arthroplasty: A Narrative Review of Third-Body Wear, Oxidative Degradation, and Cross-Linked Polyethylene Longevity

**DOI:** 10.7759/cureus.104167

**Published:** 2026-02-24

**Authors:** Sarah N Powell, Ahmed Nadeem-Tariq, Sarah Kazemeini, Catilin Wetzel, Elizabeth Theirl, Hannah Peterson, Aditya Pancholi, Dylan Fischer

**Affiliations:** 1 Department of Orthopaedics and Rehabilitation, University of Nebraska Medical Center, Omaha, USA; 2 College of Medicine, University of Nevada Las Vegas School of Medicine, Las Vegas, USA; 3 College of Osteopathic Medicine, Ohio University Heritage College of Osteopathic Medicine, Athens, USA; 4 College of Medicine, Oakland University William Beaumont School of Medicine, Rochester, USA; 5 College of Osteopathic Medicine, Touro College of Osteopathic Medicine, New York City, USA; 6 College of Osteopathic Medicine, Midwestern University, Glendale, USA; 7 Department of Orthopaedics, Naval Air Station Jacksonville, Jacksonville, USA

**Keywords:** highly crosslinked polyethylene, third-body wear, total hip arthroplasty (tha), tribological failure, tribology

## Abstract

Tribological failure remains a leading cause of revision in total hip arthroplasty (THA). Wear particles-mediated osteolysis, oxidative degradation of polyethylene, and surface damage to bearing materials contribute to mechanical loosening and long-term implant failure. This narrative review focuses on the mechanisms of tribological failure in contemporary THA and evaluates how advances in biomaterials such as highly cross-linked polyethylene, ceramic bearings, and dual-mobility constructs influence long-term implant survivorship. Highly cross-linked polyethylene has substantially reduced wear rates and osteolysis compared to conventional ultra-high-molecular-weight polyethylene (UHMWPE) but remains vulnerable to oxidative aging and fatigue cracking under certain conditions. Ceramic-on-ceramic bearings demonstrate excellent wear resistance but may present complications such as squeaking and liner fracture. Metal-on-metal bearings, once promising for low wear, have been largely abandoned due to taper corrosion and systemic metal ion reactions. Dual-mobility constructs reduce instability and dislocation but can exhibit backside wear if component positioning or polyethylene quality is suboptimal. Material and design innovations have improved tribological performance in THA, yet no bearing surface is free of trade-offs. Understanding the interaction between materials science and mechanical loading remains critical for extending implant longevity and reducing revision rates.

## Introduction and background

Introduction

Tribological failure in total hip arthroplasty (THA) refers to implant failure modes driven by friction and wear processes at the bearing surfaces [1]. Core tribological components include friction, wear, and lubrication, which influence material behavior. Over time, the articulation of hip replacement components produces wear particles that can incite biological reactions and compromise the fixation of the implant [1]. In particular, polyethylene wear debris and metal wear debris are recognized as key culprits in aseptic loosening. These microscopic particles trigger a chronic inflammatory response that leads to periprosthetic bone osteolysis and, ultimately, can lead to loosening of the prosthetic components [1]. Modern THA has evolved with alternative bearings aimed at reducing wear and thus mitigating tribological failures. Conventional metal-on-polyethylene (MoP) bearings have given way to improved materials such as highly cross-linked polyethylene (HXLPE), as well as hard-on-hard pairings like ceramic-on-ceramic (CoC) and metal-on-metal (MoM) [1]. HXLPE liners, introduced in the early 2000s, exhibit dramatically lower wear rates in simulator and clinical studies - often significantly less liner wear than conventional polyethylene - resulting in far fewer wear particles and reduced osteolysis [2]. This advance has translated to improved implant survivorship at 10-15 years, even in younger patients, as the generation of polyethylene debris is greatly minimized [3].

In summary, tribological considerations are central to the long-term success of a hip replacement. The introduction of advanced bearings has been driven by the need to reduce wear debris and its deleterious biological effects, particularly as the demands placed on implants increase and patients live longer with implants in situ. This review addresses tribological failure in THA through six main sections: fundamental wear mechanisms, polyethylene oxidative degeneration and evolution of HXLPE, HXLPE performance, CoC bearings, MoM bearings and adverse reactions, and dual-mobility (DM) systems. We will focus on how friction and wear mechanisms, as well as lubrication phenomena, contribute to tribological failure in THA and how modern design strategies attempt to mitigate these issues.

Fundamental tribological mechanisms in hip arthroplasty

Adhesive and Abrasive Wear

In THA bearings, material is gradually removed from the surfaces via several fundamental wear mechanisms. Adhesive wear occurs when two bearing surfaces in contact experience local adhesion (micro-welding) at asperities; as the surfaces slide, these junctions shear off, carrying away fragments of material as wear debris [4]. This mechanism is more likely under poor lubrication or high friction conditions, where direct metal-polyethylene or ceramic-polyethylene contact leads to transfer of material from one surface to the other [4].

Abrasive wear occurs when a harder surface or hard particle presses against a softer surface, ploughing and cutting it [4]. In a hip implant, abrasive wear can be two-body (the femoral head scratches the liner if one is significantly harder or rougher than the other) or three-body [1]. Third-body wear refers to abrasion caused by particles trapped in the articulating interface. Sources of these entrapped particles include cement debris, metallic fragments (e.g., from trunnion fretting or component microfractures), or bone fragments from acetabular reaming [1]. Once lodged between the head and cup, third-body particles act like an abrasive slurry, scratching the polished surfaces and accelerating both polyethylene and metal/ceramic wear [1]. Clinically, third-body wear is implicated in cases of unexpectedly rapid liner wear or scratches on retrieved femoral heads, underscoring the importance of clean intraoperative technique and implant designs that reduce the ingress of debris [2].

Lubrication Schemes in THA

Total hip replacements operate under mixed lubrication conditions that fluctuate with joint motion and loading. Ideally, synovial fluid would provide full fluid-film (hydrodynamic) lubrication, separating the femoral head and acetabular liner with a continuous fluid layer so that they do not make physical contact [4]. In reality, the hip often operates in the boundary regime due to its moderate sliding speeds and high loads [5]. In boundary lubrication, the fluid film is so thin that surfaces contact each other, and only a molecular boundary layer (absorbed proteins, etc.) provides slight separation [5]. In mixed lubrication, a partial fluid film exists, but some high points still rub [4]. Hard-on-hard bearings (especially CoC) can achieve higher gamma ratios and approach fluid-film lubrication under certain conditions; their extremely smooth, hard surfaces and roundness allow a thicker lubricant film at equivalent clearances [6,7]. The importance of lubrication is evident in the dramatic differences in wear rates: when operating in full fluid-film mode, hard bearings can have negligible wear, whereas boundary-lubricated conditions produce orders-of-magnitude more wear [7]. Thus, breakdown of lubrication, even transiently, is a key factor in tribological failure, as it shifts the wear mechanism back toward adhesive and abrasive interactions [8].

Classic low-friction arthroplasty used a 22 mm head to minimize sliding distance and contact area [9,10]. Contemporary practice often favors larger heads (32-40 mm) for improved stability, and the advent of HXLPE has made this possible without a proportionate wear penalty [10]. Studies have found that with highly cross-linked liners, liner wear rates remain very low and are not strongly dependent on head diameter [9]. In a series with a 5-8-year follow-up, 36-40 mm heads on HXLPE had similar liner penetration (~0.03-0.04 mm/year) to 28 mm heads, whereas older conventional polyethylene showed increasing wear with each increment in head size [2]. However, larger heads do increase the volumetric wear simply because a larger articulating surface, even wearing at the same rate, produces more total volume loss [2]. Thus, especially in young, highly active patients with decades of expected implant life, some caution is still advised with very large heads despite HXLPE’s excellent wear characteristics. 

Metal Pairings

Material pairing is another decisive factor. Hard-on-soft pairs (metal or ceramic heads on polyethylene) generate polymeric wear debris that induces osteolysis, whereas hard-on-hard pairs avoid polyethylene but introduce their own issues [10]. MoM bearings produce far fewer particles by volume, but the nano-scale cobalt-chrome debris can provoke adverse local tissue reactions and even systemic effects in susceptible patients [11]. CoC pairs generate mostly microscopic ceramic particles, which are relatively bioinert, and eliminate the osteolysis problem - yet ceramic components can suffer brittle fracture or edge-loading damage, and squeaking noises can affect patient satisfaction [10]. Newer material innovations like oxidized zirconium heads (ceramicized metal) or vitamin E-stabilized poly seek to combine advantages and further reduce wear or oxidation, though long-term data are still accruing [10].

Finally, surface roughness and finish quality have a direct impact on friction and wear. A smoother surface leads to lower frictional coefficients and less abrasive wear [12]. Modern femoral heads are polished to a mirror finish, often within the nanometers, to minimize initial wear of the liner [9]. If a femoral head or liner surface becomes scratched or roughened (e.g., by third-body particles or impingement damage), it can rapidly accelerate counter-surface wear - a scratched metal head articulating on polyethylene can increase polyethylene wear rate several-fold [12]. Ceramic heads and liners, having the hardest and smoothest surfaces, tend to maintain low roughness and thereby support better lubrication and lower wear over time. In practice, maintaining an optimally low surface roughness is critical for minimizing abrasive wear [5].

## Review

Oxidative degradation of polyethylene bearings

Conventional ultra-high-molecular-weight polyethylene (UHMWPE) has been used as the standard liner material in THA since the 1960s due to its extensive biocompatibility, wear resistance, and mechanical strength [13-15]. Its high tensile strength, impact resistance, and low coefficient of friction indicate superior wear resistance essential for long-term implant performance [15]. UHMWPE is also inert to most chemicals and has low water absorption, making it suitable for internal bodily environments while not eliciting significant immune responses [15]. However, the long-term success of UHMWPE implants is not solely dependent on their inherent material properties but also on the methods used during manufacturing and sterilization, which can significantly influence their performance and longevity [14,15].

While these implants have prominent long-term capabilities, UHMWPE is prone to oxidation after gamma radiation, which can degrade its mechanical properties and lead to wear and implant failure [15,16]. In the 1990s, gamma air-sterilized liners were found to fail prematurely due to oxidative degradation, observed in the form of rim cracking, delamination, and increased wear debris [16-18]. Conventional UHMWPE exhibits the highest oxidation indexes, especially at the rim, resulting in a reduction in molecular weight and embrittlement [19-21]. Oral et al. assessed oxidation and discovered that these lipids play a significant role in accelerating UHMPWE oxidation and compromising mechanical integrity [22].

Initial concerns about in vivo oxidation with UHMPWE have led to advances in material processing, resulting in first- and second-generation HXLPEs with improved wear performance [23]. First-generation HXLPEs undergo single high-dose irradiation and remelting or annealing [20]. These have been shown to exhibit moderate oxidation, particularly in annealed liners, accompanied by some embrittlement and damage [24]. In contrast, second-generation HXLPE undergoes sequential irradiation and annealing, exhibiting the lowest oxidation levels across all regions and showing few signs of gross mechanical degradation, which supports its improved long-term durability [1,8,20]. HXLPE initially became the standard bearing material in THA due to its reduced wear properties, which are achieved through irradiation, creating free radicals in the polyethylene [25]. HXLPE liners exhibit significant reductions in wear rates compared to conventional polyethylene, leading to a decline in osteolysis and aseptic loosening rates [11].

These acetabular liners can also undergo stabilization and thermal treatments, such as remelting or annealing, to reduce free radicals, thereby improving their overall oxidative stability [20,23]. Remelting completely melts the polyethylene, eliminating free radicals and making it initially oxidation-resistant [24]. However, remelted HXLPE has been found to exhibit a significant decrease in elastic modulus and hardness, resulting in a loss of mechanical integrity with longer implantation times due to oxidative embrittlement [6]. Despite the melted liners losing mechanical strength over time, they exhibit negligible oxidation, even after 10 years in vivo [13,14]. In contrast, annealing involves heating below the melting point to reduce free radicals while preserving mechanical properties [13]. Multiple studies have found that first-generation annealed HXLPE liners showed progressive in vivo oxidation, especially at the rim and backside [12,13]. Annealed HXLPEs, although initially improved over conventional polyethylene, may leave residual free radicals and require clinical monitoring due to the risk of degradation over time resulting from oxidation [26,27]. Derr et al. discovered that annealed liners exhibit high oxidation levels at the rim compared to remelted liners, although both have lower oxidation indexes than conventional UHMWPE [9]. Overall, remelted and annealed liners have demonstrated comparable wear performance in vivo; however, more longitudinal studies are needed to fully assess the clinical implications of their specific differences.

Further oxidative stability can be added to second-generation HXLPE liners without compromising mechanical properties by incorporating antioxidants, such as vitamin E [11]. These third-generation polyethylenes are antioxidant-stabilized by blending α-tocopherol, also known as vitamin E, into the resin during pre-processing, thereby blending it into already consolidated and irradiated polyethylene [12]. This neutralizes free radicals post-irradiation and maintains oxidative stability without remelting, all while retaining mechanical strength and fatigue resistance [2,3,16]. Retrievals and lab testing show no detectable oxidation or degradation in mid-term use, and the material maintains stable mechanical properties regardless of implantation duration [6]. Additionally, they were shown to have significantly lower wear at two years compared to UHMWPE liners, with an annual wear rate approximately four times lower than that of vitamin E-incorporated HXLPE liners [28]. These antioxidant-stabilized liners target long-term durability and ultra-low wear, enhancing stability and longevity in both conventional UHMWPE and generational HXLPE liners.

Polyethylene wear and HXLPE performance

The optimal polyethylene bearing for THA has evolved to focus on material selection that minimizes wear debris, a key catalyst for periprosthetic osteolysis - HXLPE implants have shown steady improvements in reducing wear while extending implant longevity [28].

For first-generation HXLPE implants, Kurtz et al. reported that the mean femoral head penetration rate was 0.042 mm/year compared to an average of 0.137 mm/year with UHMWPE implants, corresponding to approximately 69.3% reduction in liner wear, which translated into an 87% lower risk of osteolysis at mid-term follow-up [29-31]. Building on these outcomes, second-generation HXLPE was designed with optimized low-dose radiation doses and thermal processing, often supplemented with antioxidants such as vitamin E, further improving long-term durability [32,33]. In one study at 15-year follow-up, Moon et al. observed no signs of osteolysis in HXLPE liners, whereas 59% of conventional UHMWPE showed osteolysis [34]. Similarly, Tsukamoto et al. at long-term follow-up found at a mean of 12 years demonstrated superior survivorship with HXLPE, showing a wear rate of only 0.035 mm/year compared to 0.118 mm/year with UHMWPE and significantly fewer osteolytic lesions [35]. Even in these long-term follow-up studies, where investigators are tracking for late failure, outcomes are overwhelmingly positive for HXLPE [32,33,36]. Though some minor shortcomings were noted, HXLPE has been a major success in mitigating tribological failure and improving long-term outcomes in THA [35-37].

The performance of polyethylene liners also depends on the femoral head material and size. HXLPE, when coupled with ceramic heads, has been shown to reduce wear and the risk of third-body damage. Mertz et al. conducted a meta-analysis that showed a weighted mean wear rate of ceramic heads at 0.047 mm/year [38]. This can likely be attributed to the scratch-resistant nature of these femoral head components that minimize abrasive wear of the articulating liner [37]. In contrast, cobalt-chromium (CoCr) metal heads can become roughened by third-body particles that can increase polyethylene wear as time progresses, and these CoCr metal heads had a greater wear rate of 0.063 mm/year [37]. When considered together, high-quality CoCr vs. ceramic femoral heads in well-controlled conditions were shown to both be acceptable when used with HXLPE, with the modest differences seen likely attributed to the slightly reduced liner penetration of the ceramic heads in the liners [37,38].

Beyond materials, careful consideration of femoral head size is necessary to reduce liner wear and achieve optimal joint biomechanics. Historically, larger femoral head diameters have been associated with greater volumetric wear of conventional non-cross-linked polyethylene; however, this relationship of wear is less pronounced in HXLPE liners [39]. Lachiewicz et al. found that liner wear rates were independent of head diameter, but volumetric wear was significantly higher with larger heads [2]. Regardless of the femoral head size, component position is of equal importance as a malpositioned cup can lead to edge-loading that generates increased wear when interfacing with HXLPE liners [38]. This optimal positioning was of importance by Garcia-Rey et al., who mentioned that factors such as vertical position should be avoided to keep HXLPE wear negligible [38]. In summary, cross-linked polyethylene continues to be a vital material in joint prosthesis, as it reduces particle wear formation and greatly prolongs the lifespan of THA through its durability.

CoC bearing surfaces: ultra-low wear and unique issues

CoC THA was developed to help address the limitations of polyethylene and metal bearings. These include high wear rates and biologic reactivity. Alumina-alumina bearings in CoC hips have consistently shown to decrease osteolysis and wear compared to ceramic-polyethylene hip [40]. Although early-generation ceramics were limited by brittleness, newer-generation ceramics have reduced the risk of head fracture to 0.004% [41]. Newer generation ceramics have also retained their hallmark advantage of reduced wear. For instance, fourth-generation alumina ceramic composites have shown ultra-low wear rates of 0.01-1 μm/year for liner wear, and 0.005-2 mm^3^/year for volumetric wear, significantly lower compared to MoP and MoM [42]. These advantages have placed CoC as the most wear-resistant option for long-term implant survival.

Although ceramic debris can result in cytokine release, the biologic response is less compared to polyethylene or metal particles. CoC has been identified to result in less tumor necrosis factor-alpha (TNF-α) and prostaglandin E2 (PGE2) release [4]. Additionally, when compared to MoM, CoC surfaces have a lower volume of wear, and approximately 10 times fewer particles are released [43]. This adds to the benefit of CoC by reducing aseptic loosening and therefore prolonging the longevity of the implant. Long-term follow-up studies confirm that the wear rates of CoC hips were substantially lower than those of metal-on-HXLPE [44]. This emphasizes CoC as an ideal implant choice for improved implant longevity [43,44].

One frequently reported side effect of CoC THA is the presence of squeaking or clicking within the joint, which can affect the patient’s quality of life [44,45]. Risk factors include body mass index (BMI), patient activity level, implant position and orientation, implant design and materials, abduction angle, and stripe wear [45]. Under normal lubricating conditions, hip squeaking will not occur; however, fluid film lubrication can be disrupted through increased surface roughness or stripe wear [45]. Although often benign, it can indicate mechanical issues and should be considered a factor that may compromise the long-term success of the implant [44-46]. Walter et al. reported that noisy CoC implants wore down at a rate of 6.7 mm^3^/year, whereas the silent CoC implant wore down at a rate of 0.147 mm^3^/year [46]. There is, therefore, a strong correlation between stripe wear and squeaking.

Another potential risk is ceramic liner fracture, largely due to neck-cup impingement, or edge loading [46,47]. The importance of surgical technique, proper implant positioning, and design in reducing this potential risk cannot be overstated [47]. Although surgeons must be aware of the fracture risk, new-generation ceramics have reduced femoral head fracture rates to 0.004% and 1.12%-3.3% for liners [47].

When compared to MoP and MoM hips, CoC hips demonstrate superior performance in terms of both low wear and reduced particle generation [43,48]. Revision surgery in CoC hips, although uncommon, is often attributed to factors such as aseptic loosening, squeaking, or ceramic fracture [48].

MoM bearings: low wear vs. adverse metal reactions

MoM hip bearings were introduced with the expectation of addressing several limitations of conventional MoP implants [10,11]. By eliminating polyethylene from articulation, MoM designs aimed to reduce polymeric wear debris and reduce osteolysis and aseptic loosening that is frequently associated with MoP prosthesis [11]. In addition, their ability to incorporate larger femoral head diameters was considered advantageous for improving joint stability, lowering the risk of dislocation, and enhancing range of motion [49,50]. Despite these advantages, clinical experience demonstrated notable complications [47,48]. The sliding contact of CoCr alloys in MoM bearings generates metallic wear particles and elevates systemic metal ion levels [50,51]. These factors have been linked to biologic failures, including adverse local tissue reactions, pseudotumor formation, and early implant revision [51,52].

In the late 1990s, second-generation MoM THA were reintroduced in an effort to overcome the wear debris and osteolysis problems of earlier MoP implants [53]. These often included CoCr alloy on CoCr [53]. Laboratory hip simulator tests had shown that hard-on-hard metal bearing couples produce dramatically lower volumetric wear compared to MoP bearings, on the order of one magnitude less wear volume under standard conditions [54]. For example, one study found that modern CoCr MoM prostheses generated almost 100-fold less wear debris than a conventional MoP design [55]. This superior wear performance provided a strong rationale. By reducing wear particle load, MoM implants were believed to lower the rates of aseptic loosening and osteolysis over time [56]. Ultimately, these findings suggested that MoM designs could offer greater implant longevity compared with earlier surfaces.

Another advantage of Co-Cr alloys is their high material strength [57]. This allows the use of larger femoral head diameters (36-54 mm) with thin acetabular liners, without risk of fracture [57]. Larger heads substantially increase the jump distance and increase the range of motion before impingement to reduce overall dislocation risk [58]. These perceived advantages made MoM an attractive option for younger, active patients [54,56]. In theory, this population would benefit from a durable articulation capable of withstanding higher demands without the risk of delocation.

From a tribological perspective, MoM hip bearings operate primarily in a mixed lubrication setting [55]. This means that while a fluid film (synovial fluid) bears part of the load, there is also some direct contact between the metal surfaces [59]. Unlike hard-on-soft bearings, which often rely on full fluid-film (hydrodynamic) lubrication, the MoM surface can sustain some contact without rapid wear due to the smoothness and hardness of the surfaces [11,59]. This was one of the factors that helped distinguish MoM bearings from earlier designs.

When conditions are optimal, such as proper implant design, precise component alignment, and adequate time for a “bedding-in” period, MoM bearings may approach nearly full fluid-film lubrication [7]. During these circumstances, wear rates can stabilize at low levels, usually on the order of only a few micrometers per year [59]. A characteristic feature of MoM couples is this initial run-in phase, followed by a low steady-state wear phase [59,60]. During the run-in period (generally considered to be the first few million gait cycles or 6-12 months of use), wear is higher as the surfaces conform and microscopic roughness is polished away [60]. Simulator studies show that hard-on-hard bearings showed an order of magnitude reduction in wear volume after the initial wear-in phase compared to conventional bearings [61]. These ultra-low wear rates are roughly 5-10 times lower than those of traditional metal-on-poly bearings under comparable conditions [62]. This supports the belief that MoM bearings could extend implant longevity by reducing wear particle generation.

While pure wear of the bearing surface is low, MoM articulations introduce another mode of material degradation known as tribocorrosion [61]. This refers to the combined effect of mechanical wear and chemical corrosion [9,61]. Repetitive MoM contact not only generates particulate debris but also disrupts the protective oxide layer on the CoCr alloy to expose the metal to bodily fluids [63]. The disruptions accelerate the electrochemical corrosion at the contact interface [63]. The synergistic effect of repetitive micro-contact (wear) and electrochemical dissolution (corrosion) leads to the release of metal ions, such as divalent cobalt and trivalent chromium, into the joint space and bloodstream [64]. Even with low volumetric wear, patients with MoM hips can have elevated systemic metal ion levels due to this continuous process.

MoM performance is sensitive to implant positioning and dynamic loading conditions. If lubrication is disrupted as a result of the malposition of components, wear and corrosion can increase exponentially [65]. A common example includes edge-loading, where the acetabular cup is implanted at a steep inclination or in excessive anteversion, causing the femoral head to contact the rim of the cup [66]. This concentrates stress on a small area and produces higher wear [63]. Similarly, suboptimal component design or insufficient coverage of the femoral head can promote edge-loading and high wear.

DM bearings: tribological behavior and wear in a two-articulation system

DM cups have emerged as a widely adopted solution and now represent a key intervention in the prevention and management of hip instability [67]. A DM cup design features a metal acetabular shell, polyethylene liner, and femoral head encompassed within two articulating surfaces [65]. The inner bearing articulates with a polyethylene liner in smaller arcs of range of motion; once this range of motion is exceeded, the polyethylene liner articulates with the acetabular shell [65]. These dual-articulating surfaces reduce instability by reducing the risk of the trunnion impinging on the acetabular component and dislocating the femoral component [68]. Despite these advantages, there are concerns regarding the potential for accelerated polyethylene wear and increased debris generation due to the presence of an additional bearing [68]. In a model with HXLPE liners, the volumetric wear performance was comparable to, or even lower than, that of conventional single-bearing systems [69]. The reduced wear is primarily due to its dual-articulating interface [68,69]. This design leads to shorter sliding distance and lower frictional torque, as most motion is confined to the inner surface, minimizing overall polyethylene wear compared to single-bearing constructs [69].

The durability of dual-motion cups has been validated in hip simulation studies. A pivotal simulation evaluating polyethylene wear over five million cycles demonstrated that DM constructs produce significantly less cumulative wear compared to conventional single-bearing components [69]. The single-mobility design generated a polyethene wear of 39.6 mg/Mcy per million cycles, nearly double the 20.4 mg/Mcy generated in the DM cup [69]. These findings suggest that these new systems can leverage the wear advantage of small-head constructs without sacrificing the range of motion provided by large femoral heads because of the nuanced biomechanics.

More recent studies show a favorable clinical result with contemporary bearing methods. For example, Hannon et al. state that over 5,500 ceramic-on-HXLPE implants at mid-term follow-up had a near elimination of failure of the bearing surface [70]. In addition, HXLPE liners stabilized with vitamin E have shown promising effects in reducing femoral head penetration [71]. However, more long-term follow-up is needed to determine vitamin E’s effect on functional outcome and revision rate. In addition, recent studies showed that CoC and ceramic-on-HXLPE have similar postoperative outcomes, including revision and complications [72]. This further emphasizes the clinical relevance of HXLPE as a reliable bearing surface in contemporary THA.

## Conclusions

THA lifetime is ultimately limited by tribology. Adhesive, abrasive, third-body, and tribocorrosion processes, which are influenced by boundary/mixed lubrication, dictate debris production and the biological cascade that leads to osteolysis and looseness. Material breakthroughs have changed the risk landscape. HXLPE has decreased polyethylene wear and osteolysis, and contemporary ceramics provide ultra-low wear while adding specific failure signs. Overall durable THA hinges on minimizing debris and preserving lubrication over the implant’s life through thoughtful bearing choice, precise implantation, and vigilant postoperative monitoring.
